# The role of IFN-γ in regulation of IFN-γ-inducible protein 10 (IP-10) expression in lung epithelial cell and peripheral blood mononuclear cell co-cultures

**DOI:** 10.1186/1465-9921-8-80

**Published:** 2007-11-08

**Authors:** Maria Torvinen, Hinnah Campwala, Iain Kilty

**Affiliations:** 1 Pfizer Global R&D, Dep. Allergy and Respiratory, 500, Pfizer Ltd., Sandwich, Kent, CT13 9NJ, UK

## Abstract

**Background:**

Interferons play a critical role in regulating both the innate and adaptive immune responses. Previous reports have shown increased levels of IFN-γ, IFN-γ-inducing IL-12 and IFN-γ-inducible chemokine IP-10 in patients with chronic obstructive pulmonary disease (COPD).

**Methods:**

The present study focuses on the regulation of the IP-10 secretion in co-cultures of lung epithelial cells and peripheral blood mononuclear cells (PBMCs).

**Results:**

No IP-10 secretion was detected in cells cultured alone, whereas a significant increase in IP-10 levels was observed in epithelial cell/PBMC co-cultures. Furthermore, the results show that interactions between lung epithelial cells, lymphocytes and monocytes are needed for basal IP-10 secretion. Interestingly, we have also shown that incubation with IL-12 can induce an IFN-γ independent increase in IP-10 levels in co-cultures. Furthermore, inhibition studies supported the suggestion that different intracellular pathways are responsible of IFN-γ and IL-12 mediated IP-10 secretion.

**Conclusion:**

These studies demonstrate a novel diversity in IFN-γ/IL-12 pathways, showing that the IP-10 expression in co-cultures is regulated by multiple factors, such as intercellular interactions in addition to IFN-γ and IL-12 levels. These results may be valuable in designing novel strategies to antagonize IP-10 mediated immunological reactions and chemotactic effects on T cells.

## Background

Multiple inflammatory cells, mediators, and proteases are involved in the pathophysiology of COPD. It is characterized by chronic inflammation primarily in the small airways and lung parenchyma, with increased numbers of macrophages, neutrophils and T lymphocytes in comparison to healthy controls [1]. T helper (Th) lymphocytes can be classified into two types depending on the secreted cytokines. Th1 cells are mainly involved in cell-mediated inflammatory reactions and in development of chronic inflammatory conditions, whereas Th2 cells enhance antibody production by B cells and are prominent in the pathogenesis of allergic diseases [2,3]. A bias towards a Th1 cell profile has been hypothesized in COPD, with Th1/T cytotoxic 1 (Tc1) pattern and increased Th1 cytokine levels [1].

Th1 cells secrete IL-2, IL-12, and IFN-γ, which has been shown to regulate Th mediated immune and allergic responses by inducing Th1 differentiation. IFN-γ secretion from natural killer (NK) cells and monocytes/macrophages is likely to be important in early host defence against infection, whereas T lymphocytes become the major source of IFN-γ in the adaptive immune response [2,3].

IFN-γ-inducible protein 10 (IP-10) is induced by IFN-γ in many types of cells including monocytes and lung epithelial cells [4,5]. IP-10, also named CXCL10, is a potent chemokine for activated T lymphocytes and regulates cell proliferation, apoptosis and adhesion molecule expression [6]. Previous studies have shown that physical interactions between cells grown in co-cultures induce IP-10 secretion; between endothelial cells (EnC)/monocytes [7], EnC/alloantigen-primed T cells [8], EnC/PBMCs [9], leucocytes/synoviocytes [10] as well as human bronchial epithelial cell (BEAS-2B)/eosinophils [11]. The increased IP-10 secretion in BEAS-2B/eosinophil co-cultures was regulated by p38 MAPK and NF-kappaB activities of BEAS-2B cells, at least partly via intercellular contact [11].

IP-10 binds to a G protein coupled receptor CXCR3 that is preferentially expressed on Th1 type cells, causing chemotaxis of these cells towards this chemokine [12]. CXCR3 is also expressed by many cell types including lung epithelial cells [13,5,14] and it has been shown to be involved in epithelial cell movement via p38 MAPK and PI3K dependent signalling pathways in human airway epithelial cells (HAEC) [15]. Furthermore, HAEC have also been shown to release IP-10 as well as express CXCR3, suggesting the potential for autocrine signalling [14].

IFN-γ-inducing cytokine IL-12 is produced by many cell types including monocytes/macrophages, and neutrophils. The major actions of IL-12 are on T cells, resulting in induction of Th1 differentiation, proliferation, IFN-γ production and increased cytotoxic activity. [16] Th1 cytokine phenotype has been demonstrated in peripheral blood [17] and in lung portions removed surgically from patients with COPD [18]. Furthermore, increased IL-12 levels have been shown in patients with COPD [19,20]. Relative expression levels of IFN-γ in COPD patients are variable, with previous studies having shown an increase [19,18], decrease [21] or no change [22] in IFN-γ secretion in COPD patients compared with controls. Enhanced IP-10 secretion [23,18,24] as well as expression of the IP-10 receptor CXCR3 [23] have been demonstrated in COPD. As shown by Saetta et al. (2002), most of the CXCR3 positive cells in peripheral airways in patients with COPD were CD8+ positive T cells and produced IFN-γ [23].

The present study focuses on the regulation of the IP-10 secretion. The aim was to investigate the pathways of IP-10 secretion in a in vitro system including the cell types most likely involved in the IP-10 secretion in the lung tissue of COPD patients. Although several studies have demonstrated an increased IP-10 secretion via intercellular contact, little is known of the regulation of the Th1 IFN-γ/IL-12 pathway upon intercellular interaction between lung epithelial cells and leucocytes. Since increased activity of the IFN-γ/IL-12 pathway as well as increased levels of IP-10 in COPD is most likely due to a complex interaction between lung epithelial cells and white blood cells, we decided to investigate the role of the IFN-γ/IL-12 pathway on IP-10 secretion upon the interaction of peripheral blood mononuclear cells with two human lung epithelial cell lines, A549 (alveolar epithelial cell line), Calu-3 (bronchial epithelial cell line) in addition to primary normal human bronchial epithelial (NHBE) cells.

## Materials and methods

### Maintenance of human epithelial cell lines

Cells from a human bronchial epithelial cell line (Calu-3) and from a human alveolar epithelial cell line (A549) were used for the present studies. Both cell lines were cultured routinely at 37°C with 5% CO_2 _in Minimum essential medium (MEM) with Earle's Salts and L-Glutamine (Invitrogen) supplemented with 10% of heat-inactivated foetal calf serum (FCS) (PAA Laboratories), 1.5% sodium bicarbonate solution, (Sigma-Aldrich), 10 mM Sodium pyruvate solution (Sigma-Aldrich), 1× MEM non-essential amino acid solution (Sigma-Aldrich) and 1× Primocin (Autogen Bioclear) in cell culture polystyrene flasks with vent caps (Corning). The splitting of cell cultures was performed by replacing the medium with cell dissociation solution (Sigma-Aldrich). Both cell lines were used up to 32 passages.

### Maintenance of normal human bronchial epithelial cells

Normal Human Bronchial Epithelial Cells (NHBEs) were cultured according to the manufacturer's instructions (Cambrex, Inc.). However, during the experiment and the co-culture conditions, the NHBEs were transferred to the Minimum essential medium (MEM) with Earle's Salts and L-Glutamine (Invitrogen) supplemented with 1% foetal calf serum (FCS) (PAA Laboratories).

### Peripheral blood mononuclear cell (PBMC) Isolation

PBMCs were obtained from healthy non-smoking and smoking adult volunteers. Usage of human blood for the present studies was approved by the local ethical committee, and the informed consent of all participating subjects was obtained. The venous blood was collected into 50 ml centrifuge tubes each containing 5 ml of Hank's Balanced Salt Solution (HBSS) (Sigma-Aldrich) with 2.7% (w/v) Hepes (Sigma-Aldrich). The blood sample was diluted 1:1 with modified Dulbecco's phosphate buffered saline (PBS) without calcium chloride and magnesium chloride (Sigma-Aldrich). PBMCs were isolated with density centrifugation with ACCUSPIN™ System-HISTOPAQUE-1077 tubes (Sigma-Aldrich) at 400 g for 35 minutes at room temperature. Following centrifugation the layer containing the PBMCs (according the manufacturer's instructions) was collected, resuspended in PBS and centrifuged at 200 g for 10 minutes at room temperature. The supernatant was discarded and PBMC-rich pellet was resuspended in cell media (See above in *Maintenance of Human Epithelial Cell Lines*) with 1% FCS. A differential cell count was performed using a Beckman-Coulter Act5diff haematology analyzer to determine total cell number and the purity of the cell preparation. This method typically yields a cell suspension containing 80–95% of lymphocytes and 5–20% monocytes. The cells were resuspended in cell media with 1% FCS to 1 million white blood cells/ml and plated in 48 well cell culture polystyrene clusters (Corning) and cultured with or without A549 or Calu-3 cells.

### Conditioned media and transwell studies

PBMCs and lung epithelial cells were cultured alone in cell media with 1% FCS for 18 hours. The cells were centrifuged at 200 g for 5 minutes and the supernatant was collected, filtered with sterile 0.22 um filters and frozen at -80°C. For the experiments, PBMCs or lung epithelial cells were resuspended in the conditioned media and cultured for 18 hours.

For transwell studies, lung epithelial cells were grown to 80% confluency (approximately 1 × 10^5^) on 12 well transwell chambers (Corning). Subsequently lung epithelial cells and 5 × 10^5 ^PBMCs are co-cultured (1,5 ml/well) for 18 hours in transwell chambers separated by a filter (0.4 μM pore size) or not (as control), where-after the supernatant was collected for IP-10 and IFN-γ ELISA analysis.

### Isolation of lymphocytes from PBMCs

After resuspension in 1% FCS cell media to 1 × 10^6 ^white blood cells/ml (see above in *Peripheral Blood Mononuclear Cell (PBMC) Isolation*) the PBMCs were plated cell culture polystyrene flasks for 1 hour in 37°C with 5% CO_2_. Since monocytes attach to the plastic whereas lymphocytes stay in suspension, the supernatant was collected after 1 hour and centrifuged at 200 g for 5 minutes. A differential cell count was performed using a Beckman-Coulter Act5diff haematology analyzer to determine total cell number and the purity of the cell preparation. This method typically yields a cell suspension containing 99–100% of lymphocytes.

### Isolatation of monocytes from PBMCs with MACS

PBMCs are incubated with anti-human CD14 antibody conjugated to super-paramagnetic microbeads (Miltenyi Biotec). Labelled suspensions are passed through a depletion column in the magnetic field of a MACS separator (Miltenyi Biotec) according to the manufacturer's instructions. A differential cell count was performed using a Beckman-Coulter Act5diff haematology analyzer to determine total cell number and the purity of the cell preparation. This method typically yields a cell suspension containing 70–100% of monocytes with a contamination range between 0–30% of lymphocytes. Purity of 88%–100% of monocytes was set as acceptable range for the present studies with monocyte/lung epithelial cell co-culture studies.

### Interferon and chemokine ELISA assays

Human IP-10 and IFN-γ levels were specifically quantified with human IP-10 CytoSets™ and human IFN-γ CytoSets™ assays (Biosource). The epithelial cell lines were grown into 80% confluency before the experiments whereas the PBMCs were cultured at the density of 1 × 10^6 ^cells/ml. The cultures were performed in 48 well clusters with 0.5 ml cell media (500 000 PBMCs/well) with or without A549, Calu-3 and NHBEs. The epithelial cell lines and PBMCs were cultured either alone or in co-culture in 48 well clusters for 18 hours in cell media (see above) with 1% FCS before the ELISA assay. Pretreatments were performed with an addition of human recombinant IL-12 (100 ng/ml) (eBioscience) or human recombinant IFN-γ (0.1–10 ng/ml) (eBioscience) for 18 hours. Potential inhibitors 100 nM p38 inhibitor BIRB796, 500 nM IKK-2 inhibitor V, (Calbiochem), 100 nM beclomethasone (SigmaAldrich), 1 μM PI3 kinase inhibitor (Novartis, characterised as PIK 93 in [25]), 100 nM PDE4 inhibitor Rolipram (SigmaAldrich)), 5 μg/ml human IFN-γ antibody (Serotec MCA1554XZ) and 10 μg/ml human CD40 ab from (Serotec, MCA1590XZ), were added one hour before addition of IL-12 or IFN-γ. The chosen concentration for the inhibitors were roughly 10× IC50 from present and previous studies. All ELISA assays were performed according to the manufacturer's instructions. Maxisorp 96 well microplates (Nunc) were used for the assays and Skanwasher 300 (Skatron Instrument) was used to wash the microplates with 0.01 M PBS with 0.05% Tween 20 (pH 7.4) as the wash buffer. The results were read with microplate reader (SpectraMax 250) at 450 nm.

### Statistical analysis

All data are expressed as mean (ng/ml) ± SEM. All data were transformed into logarithmic data before the statistical analysis and compared with analysis of variance (ANOVA). The means of groups whose variances were determined to be significantly different were then compared by Bonferroni's multiple comparison test using GraphPad Prism (GraphPad Software Inc., San Diego, CA).

## Results

### Basal and IFN-γ mediated IP-10 secretion from PBMC/lung epithelial cell co-cultures

**Figure 1:**
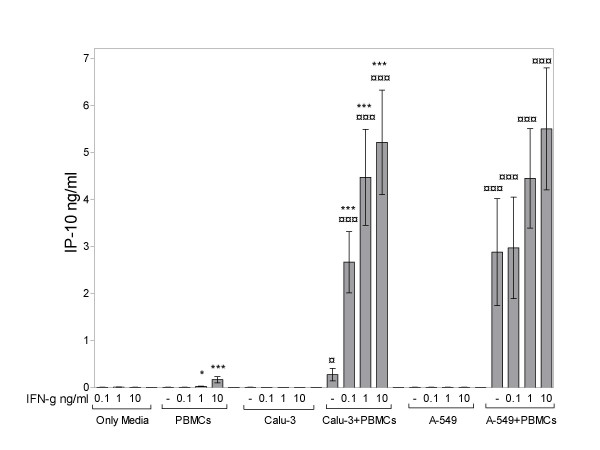
Basal and IFN-γ mediated secretion of IP-10 in lung epithelial cell/PBMC co-cultures. Data represent the mean ± SEM of 4 independent experiments, ***p < 0.001, **p < 0.01, *p < 0.05 compared to without IFN-γ treatment; ¤¤¤p < 0.001, ¤¤p < 0.01, ¤p < 0.05 for each concentration of recombinant IFN-γ treatment in co-cultures compared to both PBMCS and respective lung epithelial cell lines cultured alone, ANOVA with Bonferroni's multiple comparison test.

IP-10 levels in cell culture medium collected after 18 hours from PBMCs, Calu-3, A549 and PBMC/lung epithelial cell co-cultures were measured with ELISA. When plated alone, very little secretion of IP-10 was detected from unstimulated PBMCs and lung epithelial cell lines (Figure 1. and 2.). However, significantly increased IP-10 secretion was detected in lung epithelial cell/PBMC co-cultures (Figure 1.). The secretion from A549/PBMC co-cultures was significantly higher than Calu-3/PBMC co-cultures (p < 0.01, 0.88 ± 0.19 ng/ml and 0.22 ± 0.07 ng/ml, respectively).

**Figure 2:**
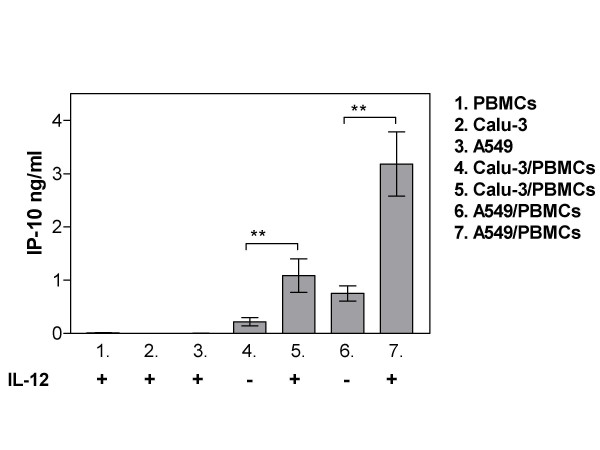
IL-12 (100 ng/ml) mediated secretion of IP-10 in lung epithelial cell/PBMC co-cultures. Data represent the mean ± SEM of 7–14 independent experiments, **p < 0.01, ANOVA with Bonferroni's multiple comparison test.

Pretreatment with recombinant IFN-γ for 18 h induced a small dose-dependent increase in IP-10 secretion in PBMCs cultured alone, whereas no detectable levels of IP-10 were found in either Calu-3 or A549 cultured alone (Figure 1). However, IFN-γ (0.1–10 ng/ml) induced a significant dose dependent increase in IP-10 secretion in lung epithelial cell – PBMC co-cultures as shown in Figure 1.

### IL-12 induces endogenous IFN-γ secretion in PBMC/A549 co-cultures

The presence of endogenous IFN-γ in supernatants collected after 18 hours from PBMCs, lung epithelial cell lines as well as in co-cultures was studied with ELISA. No detectable levels of IFN-γ were shown in either un-stimulated cells cultured alone or in co-cultures (Table 1). 18 hours incubation with recombinant IL-12 (100 ng/ml) did not induce any detectable secretion of endogenous IFN-γ in PBMCs, lung epithelial cell lines alone nor Calu-3/PBMC co-cultures (Table 1.) However, a significant increase in endogenous IFN-γ secretion was shown in A549/PBMC co-cultures after IL-12 treatment (Table 1). To establish the cell type in PBMCs interacting with the A549 cell line, secretion of IFN-γ was studied in lymphocyte/A549 and monocyte/A549 co-cultures. As shown in Table 1., lymphocytes exclusively interact with A549 resulting in a significant induction of IFN-γ secretion upon IL-12 stimulation.

**Table 1:** Secretion of IFN-γ in lung epithelial cell lines and PBMCs.

		+ IL-12
Cell media	0.002 ± 0.001	
Calu-3	0.005 ± 0.003	0.002 ± 0
A549	0.006 ± 0.002	0.003 ± 0.002
PBMCs	0.007 ± 0.003	0.007 ± 0.003
Calu-3/PBMCs	0.004 ± 0.002	0.003 ± 0.001
A549/PBMCs	0.007 ± 0.003	1.624 ± 0.36***
A549/Lymphocytes	0.010 ± 0.04	1.889 ± 0.46***
A549/Monocytes	0.018 ± 0.01	0.027 ± 0.004

Supernatants were collected after 18 hours with or without IL-12 (100 ng/ml) incubation (Data represent the mean (ng/ml) ± SEM of n = 4–10 independent experiments). Results expressed as means (ng/ml) ± SEM, n = 4–10, ***p < 0.001 compared with co-cultures without IL-12 treatment, ANOVA with Bonferroni's multiple comparison test.

### IL-12 induces IP-10 secretion in PBMC/lung epithelial cell co-cultures

18 hours preincubation with IL-12 did not modulate IP-10 secretion from cells cultured alone (Figure 2.). However, a significant increase in IP-10 secretion was observed in both Calu-3/PBMC and A549/PBMC co-cultures upon IL-12 pretreatment, as seen in Figure 2.

### IL-12 and IFN-γ co-treatment in co-cultures

The effects of IL-12 (100 ng/ml) and IFN-γ (1 or 10 ng/ml) co-treatment on IP-10 secretion was studied in Calu-3/PBMC and A549/PBMC co-cultures. No additional increase in IP-10 secretion was observed with IL-12 and IFN-γ co-treatment in A549/PBMC co-cultures compared with IL-12 or IFN-γ treatment alone (IP-10 secretion 3.7 ± 0.5 ng/ml (IL-12 100 ng/ml), 3.5 ± 7 ng/ml (IFN-γ 1 ng/ml and 3.9 ± 0.7 ng/ml (IL-12 100 ng/ml+ IFN-γ 1 ng/ml)). However, in Calu-3/PBMC co-cultures, the secretion of IP-10 induced by IL-12 pretreatment was significantly lower compared with IFN-γ induced IP-10 secretion (IP-10 secretion 1.3 ± 0.2 ng/ml (IL-12 100 ng/ml), 2.6 ± 0.4 ng/ml (IFN-γ 1 ng/ml and 3.0 ± 0.7 ng/ml (IL-12 100 ng/ml+ IFN-γ 1 ng/ml), which might be explained by the absence of IL-12 mediated induction of endogenous IFN-γ secretion when compared with A549/PBMC co-cultures (See also Table 1).

### Effects of IFN-γ antibody on IP-10 secretion

**Figure 3:**
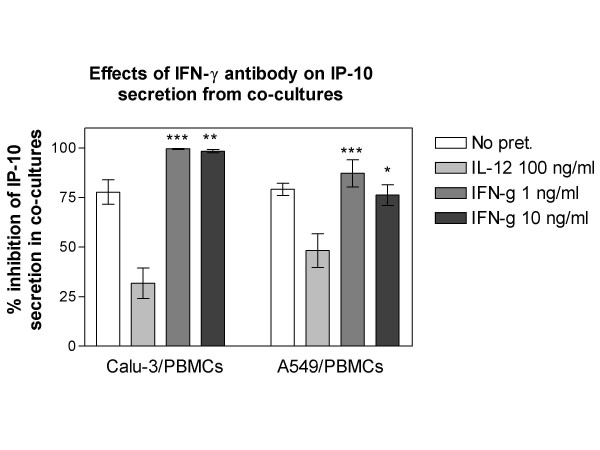
Inhibition of IP-10 secretion in lung epithelial cell/PBMC co-cultures by human IFN-γ antibody.

Treatment with 5 μg/ml IFN-γ antibody (ab) significantly inhibited the basal IP-10 secretion in both Calu-3/PBMC and A549/PBMC co-cultures (Figure 3.). The significant increase of IP-10 secretion in co-cultures mediated via recombinant (1–10 ng/ml) IFN-γ treatment was also inhibited by the IFN-γ ab treatment. However, the IL-12 induced increase in IP-10 levels was not inhibited by the IFN-γ ab, showing that at least a component of IL-12 mediated IP-10 increase is IFN-γ independent (Figure 3.).

### Conditioned media and transwell studies

**Figure 4:**
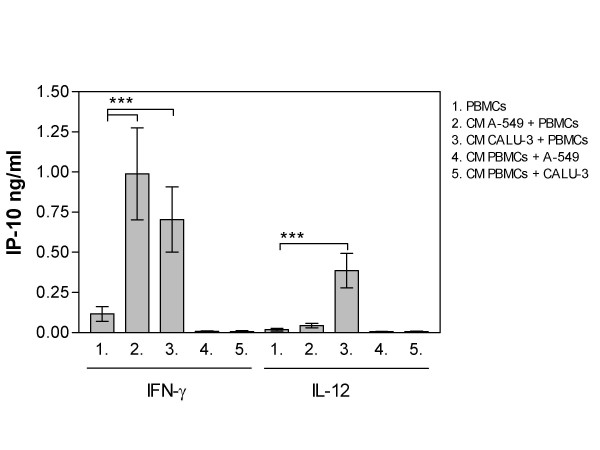
The effects of conditioned media (CM) on IP-10 secretion from cells cultured alone. The lung epithelial cells or PBMCs were cultured for 18 hours in CM simultaneously with either IFN-γ (10 ng/ml) or IL-12 (100 ng/ml) incubation. PBMCs cultured alone were used as control. Data represent the mean ± SEM of 4–6 independent experiments, *p < 0.05, **p < 0.01, ANOVA with Bonferroni's multiple comparison test.

Studies with conditioned media (CM) showed that lung epithelial cells are secreting a factor which augments IFN-γ mediated IP-10 secretion from PBMCs. PBMCs cultured with 10 ng/ml IFN-γ in CM from either Calu-3 or A549 cells induced a significant increase in IP-10 secretion compared with PBMCs cultured with IFN-γ (Figure 4.) The IP-10 is secreted by monocytes, since lymphocytes cultured with CM media from epithelial cells did not induce any IP-10 secretion (data not shown).

Furthermore, a secreted factor from Calu-3 cells augments IL-12 mediated IP-10 secretion from PBMCs. PBMCs cultured with 100 ng/ml IL-12 in CM from Calu-3 but not from A549 cells induced significant increase in IP-10 secretion compared with PBMCs cultured with IL-12 (Figure 4). IP-10 is secreted by monocytes, since lymphocytes cultured with CM media from Calu-3 did not induce any IP-10 secretion (data not shown).

No detectable levels of IP-10 were secreted by lung epithelial cells cultured in CM from PBMCs with or without IL-12 or IFN-γ treatment (Figure 4). Moreover, IL-12 treatment did not induce any detectable IFN-γ secretion from either PBMCs or A549 cells cultured in CM from A549 cells or PBMCs, respectively (data not shown).

**Figure 5:**
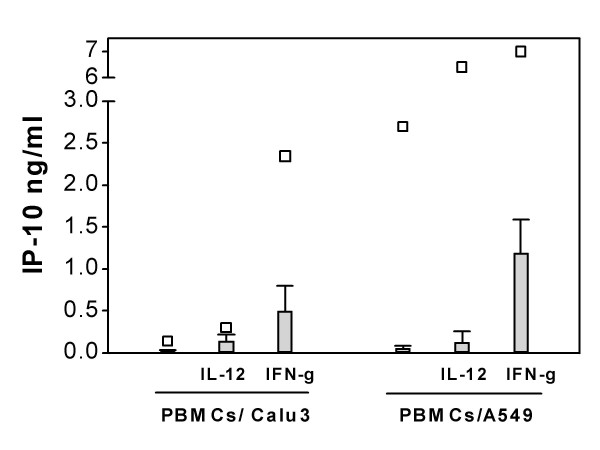
Basal, IFN-γ and IL-12 mediated secretion of IP-10 in lung epithelial cell/PBMC co-cultures cultured in transwell chambers with separating filters. Data represent the mean ± SEM of 4 independent experiments. Control (□) values are shown as IP-10 secretion in lung epithelial cell/PBMC co-cultures cultured in transwell chambers without a separating filter.

Transwell studies confirmed the results from conditioned media studies, as can be seen in Figure 5. The co-cultures were grown in transwell chambers separated (or not as control) by a filter. There is an increased IP-10 secretion in the presence of IFN-γ in co-cultures and a slight increase after IL-12 treatment (See Figure 5.). However, the basal, IFN-γ and IL-12 induced secretion of IP-10 in co-cultures is significantly decreased when separated with filter as compared to controls (Figure 5). These results confirm the results from conditioned media studies but also show that cell-cell interactions are likely to play an important role in IP-10 secretion in PBMCs/lung epithelial cell co-cultures.

However, endogenous IFN-γ secretion in lymphocyte/A549 co-cultures after IL-12 treatment was high (0.90 ± 0.45, mean (ng/ml) ± SEM, n = 3) even with separating filter, showing that although a co-culture of lymphocytes and A549 cells is necessary for the secretion of IFN-γ, no actual cell-cell contact is required.

### Studies with monocyte or lymphocyte/lung epithelial cell co-cultures

Neither basal nor IFN-γ mediated secretion of IP-10 was observed in A549/lymphocyte or Calu-3/lymphocyte co-cultures (data not shown) Treatment with IL-12 did not increase IP-10 levels in lymphocyte-Calu-3 co-cultures and only modest IP-10 secretion was observed in lymphocyte/A549 co-cultures (0.006 ± 0.005 and 0.103 ± 0.209 ng/ml, respectively, n = 3).

Furthermore, low basal increase of IP-10 secretion was observed in both Calu-3/monocyte and A549/monocyte co-cultures (0.2 ± 0.1 and 0.3 ± 0.07 ng/ml, respectively, n = 5) compared with Calu-3/PBMCs and A549/PBMCs co-cultures (1.0 ± 0.3 and 3.0 ± 1.0 ng/ml, respectively, n = 5), showing that the interactions between all three cell types, monocytes, lung epithelial cells and lymphocytes, are crucial for the basal secretion of IP-10. However, treatment with recombinant IFN-γ increases IP-10 secretion in monocyte/lung epithelial cell co-cultures in the absence of lymphocytes (Calu-3/monocyte and A549/monocyte co-cultures (2.6 ± 0.5 and 2.7 ± 0.7 ng/ml, respectively, n = 5).

### Inhibition of IP-10 secretion from PBMC/lung epithelial cell co-cultures

**Figure 6:**
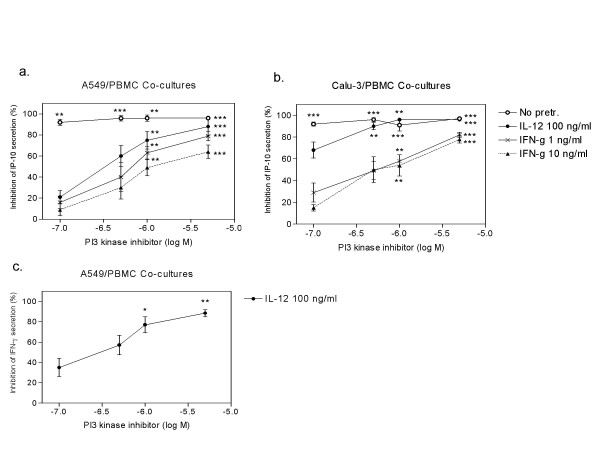
The dose dependent inhibition of IP-10 secretion by PI3 kinase inhibitor in Calu-3/PBMCs (A.) and A549/PBMCs (B.) co-cultures with or without IFN-γ and IL-12 treatment. The dose dependent inhibition by PI3 kinase inhibitor of IL-12 mediated IFN-γ secretion in A549/PBMCs co-cultures is seen in (C.) Data represent the mean ± SEM of 4–7 independent experiments, *p < 0.05, **p < 0.01, ***p < 0.001, ANOVA with Bonferroni's multiple comparison test.

P38 inhibitor BIRB796, IKK-2 inhibitor V, beclomethasone, PDE4 inhibitor rolipram and PI3 kinase inhibitor strongly and significantly inhibited basal IP-10 secretion from PBMC and lung epithelial cell co-cultures (Table 2 and Figure 6.).

**Table 2:** Inhibition of IP-10 secretion in co-cultures. The effects of 100 nM p38 inhibitor BIRB-796, 100 nM beclomethasone, 500 nM IKK-2 inhibitor V, 100 nM PDE4 inhibitor rolipram, 1 μM PI3 kinase inhibitor and human 10 μg/ml CD40 antibody on secretion of IP-10 from co-cultures.

	Calu-3/PBMC			A549/PBMC		
	
	Basal	IFN-γ	IL-12	Basal	IFN-γ	IL-12
BIRB-796	84 (± 7) **	18 (± 6)	85 (± 6) **	94 (± 2) ***	14 (± 6)	21 (± 8)
IKK-2 inh. V	75 (± 11) *	3 (± 1)	60 (± 13)	90 (± 5) *	4 (± 6)	17 (± 5)
Beclomet.	97 (± 1) ***	-7 (± 6)	94 (± 2) ***	89 (± 8) **	1 (± 4)	43 (± 14) *
Rolipram	62 (± 13) *	8 (± 2)	82 (± 10) *	77 (± 11) **	17 (± 15)	0.6 (± 4)
PI3 kin. Inh.	91 (± 5) ***	54 (± 9)	96 (± 3) **	96 (± 3) ***	50 (± 7)	77 (± 8)
CD40 ab	-14 (± 14)	-2 (± 7)	-5 (± 25)	19 (± 10)	-9 (± 12)	-12 (± 10)

Data represents the percentage of inhibition, mean (SEM). ***p < 0.001, **p < 0.01, *p < 0.05, ANOVA with Bonferroni's multiple comparison test.

The IFN-γ (10 ng/ml) mediated IP-10 secretion in both A549/PBMC co-cultures and Calu-3/PBMC co-cultures was dose dependently inhibited by the PI3 kinase inhibitor (Figure 6). In contrast, IL-12 mediated secretion of IP-10 in Calu-3/PBMC co-cultures was significantly inhibited by BIRB796, beclomethasone and rolipram (see Table 2). However there is a clear difference with the A549/PBMC co-culture, whereby IL-12 mediated IP-10 secretion was partially inhibited by beclomethasone and PI3 kinase inhibitor only (see Table 2). Human CD40 antibody (10 μg/ml) did not have any effects on IP-10 secretion in co-cultures (Table 2).

### Inhibition of IFN-γ secretion from PBMC/A549 co-cultures

IL-12 mediated IFN-γ secretion in PBMC/A549 co-cultures was inhibited significantly by p38 inhibitor BIRB796, beclomethasone, and PI3 kinase inhibitor as seen in Table 3 and Figure 6.

**Table 3:** Inhibition of IFN-γ secretion in co-cultures. The effects of 100 nM p38 inhibitor BIRB-796, 100 nM beclomethasone, 500 nM IKK-2 inhibitor V, 100 nM PDE4 inhibitor rolipram, 1 μM PI3 kinase inhibitor and human 10 μg/ml CD40 antibody on secretion of IP-10 from co-cultures.

	A549/PBMC + IL-12
BIRB-796	99 (± 0) ***
IKK-2 inh. V	11 (± 9)
Beclomet	98 (± 0) ***
Rolipram	26 (± 7)
PI3 kin. Inh.	77 (± 7)*
CD40 ab	26 (± 11)

Data represents the percentage of inhibition, mean (SEM). ***p < 0.001 and *p < 0.05, ANOVA with Bonferroni's multiple comparison test.

### IP-10 secretion in PBMC/NHBE co-cultures

**Figure 7:**
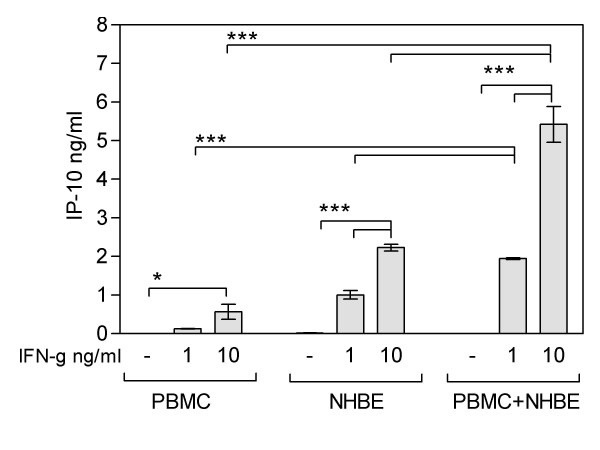
Basal and IFN-γ mediated secretion of IP-10 in NHBE/PBMC co-cultures. Data represent the mean ± SEM of 4 independent experiments, ***p < 0.001, *p < 0.05 with ANOVA.

As shown in Figure 7. no basal IP-10 secretion was observed in PBMC/NHBE co-cultures. IFN-γ treatment significantly increased IP-10 secretion from NHBEs and PBMCs cultured alone. Interestingly, IFN-γ mediated IP-10 secretion was significantly increased in co-cultures compared to the PBMCs and NHBEs cultured alone in agreement with the A549/PBMC and Calu-3/PBMC co-cultures (See Figure 7).

## Discussion

IP-10 was initially identified as IFN-γ inducible protein [26], which was shown to be a potent chemokine for Th1 cells. Its receptor CXCR3 is predominantly expressed by Th1 cells [18] but expression has also been shown in many other cell types including lung epithelial cells [5]. Increased levels of both IP-10 and CXCR3 have been shown in patients with COPD, and subsequently this chemokine has been suggested to be involved in the inflammatory process underlying COPD [23].

**Figure 8:**
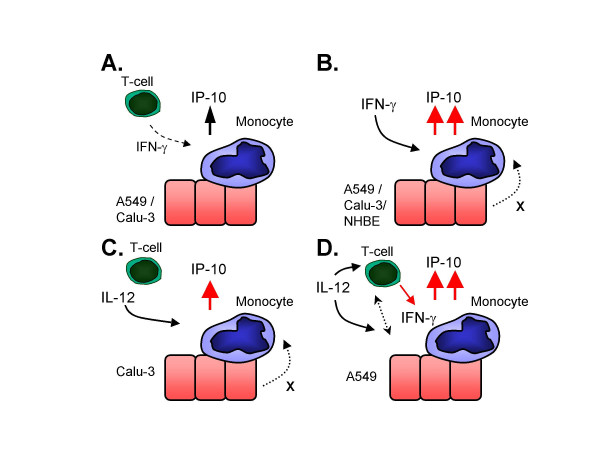
In summary, basal IP-10 secretion is induced by monocyte-epithelial cell interactions, with a presence of lymphocytes, most likely to provide a source of IFN-γ. The interaction of monocytes and lung epithelial cells are made by direct cell-cell contact. (A). Addition of recombinant IFN-γ induces strong IP-10 secretion in co-cultures even in absence of lymphocytes. Moreover, a secreted factor from lung epithelial cells augments the IFN-γ mediated secretion of IP-10 from monocytes (B). Addition of recombinant IL-12 induces IFN-γ independent IP-10 secretion in Calu-3/PBMC co-cultures which cannot be blocked by IFN-γ antibodies. Moreover, no detectable IFN-γ is present and Calu-3 cells secrete a factor which augments IP-10 secretion from monocytes in response to IL-12 (C). Addition of recombinant IL-12 induces IP-10 secretion both by inducing IFN-γ secretion from lymphocytes and by an IFN-γ independent pathway, which cannot be blocked by IFN-γ antibodies (D).

The aim of the present studies was to examine the effects of lung epithelial cells/PBMCs interaction on IP-10 secretion. We used PBMCs from both non-smoking and smoking volunteers since COPD is a smoking related disease. However, no differences were found in IP-10 secretion from PBMCs between non-smokers and smokers (results not shown). This is likely due to the fact that all volunteers used in the present study are healthy. However, at the present studies we characterize the complex interaction between PBMCs and lung epithelial cells on the regulation of IP-10 secretion by IFN-γ/IL-12 pathways. No basal secretion of IP-10 was observed in either cell type cultured alone, however, a significant increase of basal IP-10 secretion was observed in PBMC/lung epithelial cell co-cultures. The IP-10 secretion was found to be due to a specific interaction between monocytes and lung epithelial cells via cell-cell contact (Figure 8a), since no basal IP-10 secretion was detected in PBMC/lung epithelial cell transwell co-cultures. Surprisingly, no IP-10 secretion was observed in monocyte/lung epithelial cell co-cultures in the absence of lymphocytes. Since addition of recombinant IFN-γ could restore the elevated IP-10 secretion in monocyte/lung epithelial cell co-cultures, the significance of the lymphocytes in co-cultures is most likely to the source of endogenous IFN-γ (Figure 8b). A similar mechanism might also be involved in EnC/PBMC co-cultures studied by Raju et al. (2003) demonstrating that the basal secretion of IP-10 from EnC/PBMC co-cultures is IFN-γ dependent [9]. Therefore, it is likely that the increased amounts of leucocytes in lung tissue in COPD patients interact with several cell types including lung epithelial cells as well as endothelial cells in a similar manner increasing IP-10 secretion.

However, there are crucial differences in the IP-10 secretion from different types of co-cultured cells. In our study CD40 is not involved in the cell-cell interaction dependent basal IP-10 secretion, whereas CD40 has been reported to mediate IP-10 secretion in EnC/monocyte co-cultures [7]. Moreover, antibodies against ICAM, CD11b and CD18b have been used to show the importance of these proteins in leucocyte/synoviocyte IP-10 induction [10].

IP-10 is classically induced by IFN-γ, however, in the present studies no detectable basal secretion of IFN-γ was observed in the co-cultures. Nevertheless, antibodies against IFN-γ blocked the IP-10 secretion from co-cultures, suggesting that low levels of endogenous IFN-γ, undetectable with ELISA, are present in co-cultures The detection range for the IFN-γ ELISA is from ~0.015–1 ng/ml. The lowest detectable concentration would not be able to stimulate IP-10 secretion in PBMC cultures, since we did not detect any IP-10 secretion with 0.1 ng/ml IFN-γ (Figure 1). However, as shown in Figure 1, addition of 0.1 ng/ml IFN-γ strongly augments basal IP-10 secretion in Calu-3/PBMC co-cultures, which did not secrete any detectable levels of endogenous IFN-γ, suggesting that even a very low concentration of endogenous IFN-γ can induce strong IP-10 secretion when there are direct cellular interactions between monocyte and lung epithelial cells. The increasing concentrations (0.1–10 ng/ml) of IFN-γ resulted in a dose dependent increase in IP-10 secretion in co-cultures (Figure 1).

As previously described, IP-10 is specifically secreted by the monocytes in PBMCs. Interestingly, monocytes cultured in the conditioned media from either epithelial cell line, together with recombinant IFN-γ, induce significant increase in IP-10 secretion. These results suggest that a secreted factor from epithelial cell lines is at least partially responsible for the IFN-γ mediated IP-10 secretion in co-cultures. A recent study by Boulday et al. (2006) reported that vascular endothelial growth factor (VEGF) augments the IFN-γ mediated secretion of IP-10 in endothelial cells [27]. Interestingly, Koyama *et al *[28] show that A549 epithelial cells constitutively express high levels of VEGF and that this is augmented by IFN-γ. Whilst our studies confirm the high constitutive VEGF secretion (data not shown) neither human recombinant VEGF nor VEGF inhibitors had any effects on IP-10 secretion from monocytes. These data suggest that there are distinct soluble factors governing the IP-10 response in endothelial versus epithelial cells. The secreted factor from lung epithelial cells might be a growth factor, interleukin or interferon, since previous studies have shown an inducible expression of IP-10 in a wide variety of tissues and cells under the influence of stimuli including interferons, interleukins, lipopolysaccharide, tumor necrosis factor-α, platelet derived growth factor, and hypoxia [6].

IL-12 is a classic IFN-γ inducing cytokine, which induced secretion of endogenous IFN-γ in A549/PBMC co-cultures due to a specific interaction between lymphocytes and A549 cells IL-12 also induced an increase in IP-10 secretion in A549/PBMC co-cultures, potentially partly due to endogenous IFN-γ signalling. The IL-12 mediated induction of IFN-γ and IP-10 secretion in A549/PBMC co-cultures is via intercellular contact as this was only observed in co-cultures and not in transwells or conditioned media studies. Interestingly, IFN-γ antibody pre-treatment only partially inhibited IL-12 mediated IP-10 induction, suggesting that there may be both IFN-γ dependent and independent IP-10 induction pathways.

In contrast to A549/PBMC co-cultures, IL-12 significantly increased IP-10 secretion in Calu-3/PBMC co-cultures in the absence of any detectable increase in IFN-γ levels (Compare Figures 8c and 8d). Moreover, the IL-12 mediated IP-10 secretion was shown to be IFN-γ independent, since it could not be inhibited by the IFN-γ ab in Calu-3/PBMC co-cultures. This IL-12 mediated IP-10 secretion is likely to be mediated at least in part via a secreted factor from Calu-3 cells as it is maintained in conditioned media and transwell studies.

To further probe the signalling pathways involved in modulating IP-10 expression in the epithelial cell/PBMC co-cultures, we investigated the pharmacological effect of a number of signal transduction pathway inhibitors on this model. Present studies suggest that there are at least two pathways by which IP-10 can be induced which are either IFN-γ dependent or IL-12 dependent.

IFN-γ dependent IP-10 expression was sensitive to PI3K inhibitors and independent of signalling via IKK-2, p38 or PDE4. Interestingly, whilst corticosterioids are frequently prescribed for lung inflammation, they again did not modulate IFN-γ induced IP-10 expression in this system. As IFN-γ signals via a JAK-STAT1 pathway [2], resistance to these inhibitors would be expected, but the role of PI3K is very exciting. The PI3 kinase inhibitor PIK-93 used in the present studies targets several PI3 kinases and has high potency for the class I PI3 kinases p110α as well as p110γ. [25] The development of subtype specific inhibitors will help identify which subtype of PI3 kinase is responsible for the increased IP-10 expression in co-cultures. Consistent with these results, it has been reported that the non-selective PI3K inhibitor wortmanin can also inhibit IFN-g mediated IP-10 production from endothelial cells [27]. These studies suggest that the development of PI3K inhibitors may represent a novel anti-inflammatory treatment for COPD, as they will inhibit a pathway not modulated by current therapies.

In contrast, IL-12 mediated IP-10 induction was sensitive to each of the inhibitors tested, except antibodies to IFN-γ. This provides further evidence therefore, that there are at least two pathways for IP-10 induction, with the latter being dependent upon the classical inflammatory pathways, NFκB and p38 MAP kinase, as well as cAMP. Moreover, the IL-12 signalling cascade has previously been shown to be sensitive to dexamethasone, [29] and the present studies show that the IL-12 mediated induction of IP-10 in co-cultures is modulated by corticosteroids, which may contribute to the efficacy of these agents in treatment of respiratory inflammation.

The differences in IL-12 mediated IP-10 secretion between Calu-3/PBMC and A549/PBMC co-cultures were also evident in the inhibitor studies. Inhibition of IP-10 secretion in A549/PBMC co-cultures was only effectively inhibited by PI3K inhibitors and partially inhibited by dexamethasone. As these co-cultures were shown to endogenously express IFN-γ (Table 1), this would suggest that most of the drive to induce IP-10 was due to the IFN-γ JAK-STAT1 pathway, in addition to some residual signalling via a steroid sensitive pathway. In contrast, all inhibitors used in the present study strongly inhibited IP-10 secretion in Calu-3/PBMC co-cultures, suggesting IFN-γ signalling is not required for induction of IP-10. These differences might reflect the differences in bronchiolar vs alveolar lung epithelial tissue, which would have to be taken into account in design of novel inhibitors blocking the abnormally high IP-10 secretion in lung tissue of COPD patients.

In addition to the human lung epithelial cell lines, we also used the primary human epithelial cultures for the key experiments. In contrast to A549 and Calu-3, NHBEs cultured alone secrete IP-10 if pretreated with IFN-γ. Consistent with this result Sauty et al. reported that pre-treatment with IFN-γ induces IP-10 secretion in NHBEs but not in A549 cells [5]. However, in agreement with the results from A549/PBMCs and Calu-3/PBMCs co-cultures, significantly increased IFN-γ mediated IP-10 secretion was observed from NHBE/PBMC co-cultures compared with NHBEs or PBMCs cultured alone. This demonstrates a significant increase in IFN-γ mediated IP-10 secretion in PBMCs co-cultured with all lung epithelial cell lines as well as the primary bronchial epithelial cells used in the present study. These results indicate that PBMC-lung epithelial cell interactions are strongly promoting IP-10 secretion in response to IFN-γ, thereby attracting more lymphocytes to lung tissue and support the use of the A549 and CALU-3 cell lines as a model of the primary cell system. As example, application of cigarette smoke extract in the leucocyte-lung epithelial cell co-cultures or to the conditioned media is likely to provide an interesting additional in vitro model for COPD.

Since IP-10 is a potent chemoattractant for T cells, the suppression of the increased IP-10 levels in lung tissue of COPD patients may reduce the lung inflammation characteristic of this disease. Increasing IP-10 levels will cause a positive feedback loop attracting more T cells to the peripheral airways, in turn increasing IFN-γ secretion. Establishing a method to inhibit this positive feedback loop may be profitable in suppressing the inflammatory process underlying COPD. Barnes et al. (2004) suggests that T cell inhibitory strategies, such as the use of immunosuppressant's, might be effective in COPD, although side effects, such as increasing the risk of bacterial infection, is of particular concern. Inhibition of IFN-γ signaling may provide another approach [1]. As shown in the present study, basal IP-10 secretion in co-cultures is blocked with all inhibitors used, representing both current and experimental therapies for respiratory disease. However, in the presence of IFN-γ which is secreted by T cells in peripheral airways IP-10 secretion is only inhibited by inhibitors of PI3K. This in vitro model may represent the environment in the peripheral airways of COPD patients which contain a large number of Th1 T cells, and suggest that IP-10 mediated inflammation is not being addressed with current respiratory therapies such as corticosteroids in these patients. However, this pathway was modulated by non-isozyme selective PI3K inhibitors in this model. A number of Pharmaceutical companies are developing PI3K inhibitors and these results complement an emerging body of data that suggest they may also have utility in treating the inflammation associated with COPD [30].

## Conclusion

IP-10 secretion is a potent chemokine for CD8 T cells and its expression is induced when circulating monocytes, T cells and epithelium are in close proximity. Moreover, expression of this chemokine is induced by signaling molecules such as IFN-γ and IL-12 known to be expressed in COPD. Therefore, it is tempting to speculate that therapies targeted at decreasing the levels of IP-10 in peripheral airways of COPD patients may have therapeutic benefit in the management of this disease. In the present studies we demonstrate a complex interaction between monocytes, lymphocytes and lung epithelial cells resulting in IP-10 secretion via multiple pathways. Furthermore, inhibition studies supported the suggestion that different intracellular pathways are responsible for IFN-γ and IL-12 mediated IP-10 secretion. These results may provide novel strategies for investigating means by which to modulate IP-10 mediated secretion and chemotactic effects on T cells.

## Abbreviations

CM - Conditioned media; 

COPD - Chronic obstructive pulmonary disease; 

EnC - Endothelial cells; 

IP-10 - IFN-γ-inducible protein 10

## Competing interests

All experiments performed in this study are supported by Pfizer Ltd. Authors declare that they do have no competing interests.

## Authors' contributions

MT carried out experiments, performed statistical analysis, participated in the design of study and helped drafting of the manuscript; HC carried out experiments and performed statistical analysis, IK participated in the design of study and helped drafting of the manuscript. All contributors approved the final manuscript.
